# Identification and quantification of change in Australian illicit drug markets

**DOI:** 10.1186/1471-2458-6-200

**Published:** 2006-08-03

**Authors:** Stuart Gilmour, Inge Koch, Louisa Degenhardt, Carolyn Day

**Affiliations:** 1 National Drug and Alcohol Research Centre, University of New South Wales, Sydney, NSW 2052, Australia; 2 Department of Statistics, University of New South Wales, Sydney, NSW 2052, Australia; 3 National Centre in HIV Epidemiology and Clinical Research, University of New South Wales, Sydney, NSW 2052, Australia

## Abstract

**Background:**

In early 2001 Australia experienced a sudden reduction in the availability of heroin which had widespread effects on illicit drug markets across the country. The consequences of this event, commonly referred to as the Australian 'heroin shortage', have been extensively studied and there has been considerable debate as to the causes of the shortage and its implications for drug policy. This paper aims to investigate the presence of these epidemic patterns, to quantify the scale over which they occur and to estimate the relative importance of the 'heroin shortage' and any epidemic patterns in the drug markets.

**Method:**

Key indicator data series from the New South Wales illicit drug market were analysed using the statistical methods Principal Component Analysis and SiZer.

**Results:**

The 'heroin shortage' represents the single most important source of variation in this illicit drug market. Furthermore the size of the effect of the heroin shortage is more than three times that evidenced by long-term 'epidemic' patterns.

**Conclusion:**

The 'heroin shortage' was unlikely to have been a simple correction at the end of a long period of reduced heroin availability, and represents a separate non-random shock which strongly affected the markets.

## Background

In January 2001 injecting drug users (IDUs), law enforcement and treatment agencies noticed a sudden and unexpected reduction in the availability of heroin in New South Wales (NSW), Australia [1,2]. The reduction in heroin supply had a significant effect on other illicit drug markets, as IDUs initially increased their consumption of cocaine and amphetamines [3]. This phenomenon has become known as the Australian 'heroin shortage' (or 'heroin drought'). The shortage was associated with significant changes in a variety of indicators of criminal activity [4], reductions in the prevalence of heroin and other drug overdoses [5], and in IDU treatment-seeking activity [6]. The wide attention which this 'heroin shortage' gained and its impact on a wide range of indicator and sentinel data sets reinforced the significance of the phenomenon (see for example [7]).

There has, however, been some debate as to whether or not the 'shortage' represents a sudden and unexpected reduction in the availability of heroin or the end of a widespread 'glut' of heroin experienced in major Australian markets in the two years preceding the shortage [8]. Dietze and Fitzgerald (2002) have argued that the late 1990s, when heroin markets in Australia were rapidly expanding, represented a heroin "glut" and that the "shortage" was simply a return to levels of availability that prevailed before this heroin "glut". While acknowledging that there had been a sharp *relative *reduction in heroin supply, they suggested that its magnitude had been exaggerated by drug market monitoring put in place in the mid 1990s. Although there has been considerable insight into the probable causes of the shortage [9], the debate is in one sense academic, since it is not clear whether it could occur again at some future time, but the question remains important for policy discussion and policy making regarding Australian illicit drug markets. The question of whether a sudden collapse is a natural part of the final phase of an 'epidemic' cycle in illicit drug markets, or whether such a sudden collapse must be sparked by independent events is crucial to our understanding of the driving forces in the illicit drug market. Information about such a phenomenon also enables drug policy makers to be better prepared for future collapses (if they are inevitable) or to better isolate their causes (if they are caused by independent events which could potentially be manipulated for the purposes of supply reduction).

In this paper we investigate this important question using two statistical methods: principal component analysis and SiZer analysis. Principal component analysis [10] enables us to contrast different periods of time in illicit drug market data, while the SiZer analysis [11] identifies statistically significant periods of change over different time scales in key data series. By integrating these two analyses we can answer the question of whether or not epidemic cycles such as those described by Dietze and Fitzgerald exist in Australian illicit drug markets. Furthermore we identify the scale over which significant changes occur in data series which show evidence of epidemic behaviour, and compare the relative importance of epidemic and non-random shock patterns to the long-term development of the illicit drug markets.

## Methods

### Data

Principal component analysis and the SiZer were applied to 17 key indicator data series from NSW (Table 1). These data series were identified by key informants and prior analysis as being associated with drug markets [12]. The data series represent counts of all events in NSW, and there were no missing values. The data series were collected over 66 months from January 1997 to June 2002. These data series were collected prospectively at their administering agencies, but were not used to identify the time of onset of the heroin shortage, since there is a 6–12 month delay between collection of the data and its analysis. If the range of different data series varies considerably, it is standard practice [10] to scale or standardise the original data. This was done by subtracting from each series its own long-term mean, and dividing by its own series-specific standard deviation.

### Principal component analysis

PCA explains structure in data by identifying those directions in multivariate data sets which contain the most relevant information. This process often reveals relationships in data that were not previously suspected, allowing interpretations that would not otherwise be possible. The directions are ordered by the amount of variance they explain, starting with the direction that explains the largest single contribution to variance. The method is classical in origin ({Lattin, 2003 #264} [13]). In essence, PCA extracts the statistically uncorrelated data directions as the eigenvectors of the covariance matrix of the original data, and orders them in descending order of magnitude of their corresponding eigenvalues. The extent to which each variable in the original data contributes to a particular rotated direction is determined by the components of these eigenvectors, called the 'loadings'. Variables whose loadings are of a different sign in the same eigenvector can be said to be contrasted against one another in the rotated directions calculated by PCA. For the 17 key indicator data sets, where the dimensions correspond to time points – here subsequent months – the loadings represent periods of time with constant behaviour from clusters or runs of loadings similar in sign and magnitude.

### Calculation of principal components

PCA was carried out in Matlab 6.1. To calculate principal components we treated the data as a multivariate data set of 66 components or *dimensions *and 17 *observations*, i.e. we have n = 17 and p = 66. With the data in this format, the months form the 'variables' on which we wish to identify the loadings, and each data series represents one observation of these 66 variables. This enables us to assign the months into partitions according to their loadings. Because PCA cannot identify more eigenvectors than there are observations, we obtain at most 17 eigenvectors. To obtain an interpretation based on the principal component directions, we examine the loadings (the values of components in the eigenvectors) for 'runs', 'jumps' and 'isolated points'. Isolated points if large indicate outliers in a particular component. Runs are (uninterrupted) sequences of values along the components which do not differ much from their neighbouring points, and therefore indicate that there is not much change along these components. Jumps between successive points signify change. Runs separated by jumps give rise to clusters which describe the relationship of the data. Because these clusters were not identified using a measure of distance per se, we refer to them as *partitions *for the remainder of this paper.

### The SiZer

SiZer (Significance of Zero crossings of the derivative) is a method for the statistical analysis of rates of change in a data series over a range of different scales. SiZer applies a family of local linear smoothers (with Gaussian kernel weights) to the data series, varying the 'smoothing parameter' or 'bandwidth' continuously and presenting the results for discrete points in the range of possible bandwidths. In this analysis, a larger bandwidth represents a longer time frame over which changes in the series were studied. The rate of change (derivative) of each kernel density estimator is calculated, and a plot created at each bandwidth which indicates pictorially whether the derivative is significantly different from zero at each point on the chart. If the derivative is significantly different and increasing, the region over which this occurs is coloured blue; if significantly different and decreasing, the region is coloured red; and if not significantly different to zero (i.e. level) the region is coloured purple. Regions with insufficient amount of data are coloured grey. A two-dimensional plot shows the regions of increase and decrease as a function of time (the x-axis) and bandwidth (the y-axis) (see, for example, Figure 5). SiZer tests of the significance of slopes over the entire data set are adjusted for the additional type I error in multiple tests {Chaudhuri, 1999 #243}. Serial dependence is not incorporated into the SiZer model as presented here, but the authors have previously estimated that there is only limited serial dependence in the heroin deaths series {Degenhardt, 2004 #245}. Serial dependence in the cocaine possession offences data has been estimated as a positive AR(1) term that will only inflate variance of regression parameter estimates by about 11% {Degenhardt, 2005 #233}. Serial dependence will therefore have a limited effect on the significance of results presented here.

SiZer was applied to the heroin overdose deaths and the cocaine possession offences data using software available from Marron et al [11] and implemented in Matlab 6.1. These are two iconic data series representing major structural phenomena in the illicit drug markets which have previously been studied using time series methods [14,5]. Although ideally cocaine markets would be better studied using measures of the health effects of cocaine which are independent of police activity, no such health indicators exist, and state wide data on possession offences were deemed the next best data series for objective assessment of cocaine market activity [15].

## Results

**Figure 1:**
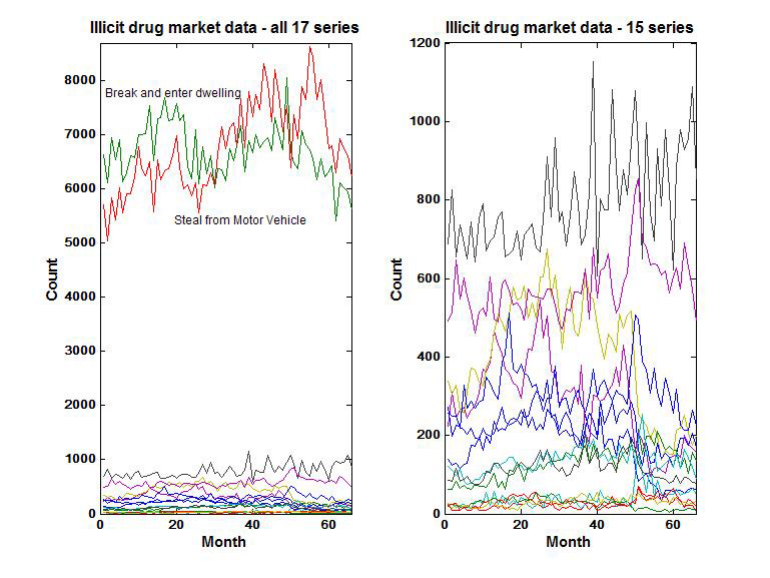
Parallel coordinate plots of complete illicit drug market data.

The entire data set is shown in Figure 1 in parallel plot form (i.e. as a combined plot of all 17 data series with the x-axis as the months, and different series displayed in different colours) [16]. Here each line corresponds to a single data series, and each data series is distinguishable by a unique colour. The left-hand plot contains 17 data series and shows a clear pair of outlier series (*break and enter dwelling *and *steal from motor vehicle*) while the right hand plot shows the dataset with these two series removed. The remaining 15 data series clearly span a range of scales, and excluding the two outlying series will not remove the dependence of the variance structure on individual series, indicating that it is better to standardise all data series than to remove outliers.

**Figure 2:**
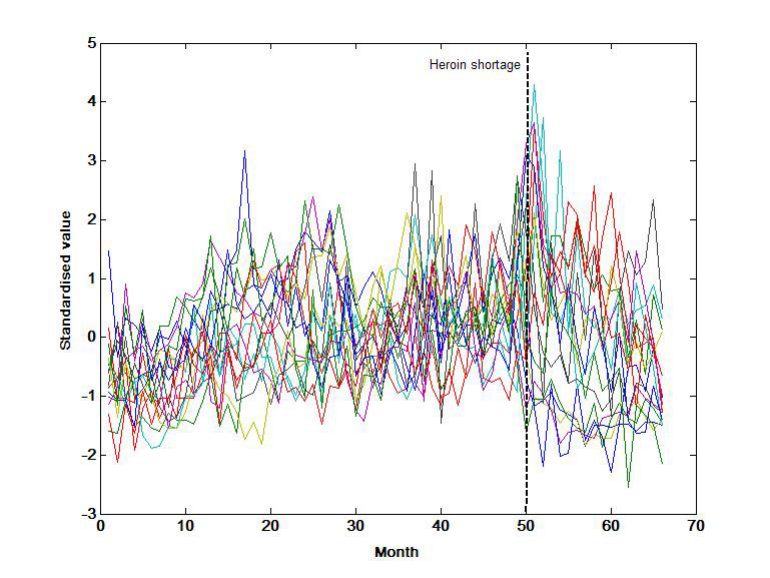
Parallel coordinate plot of the standardised data.

The standardised data is shown in Figure 2, and shows some evidence of a peak or change in behaviour at about month 50 (the widely accepted onset point of the heroin shortage [17]) and limited curvature across the plot. In this form the data is difficult to interpret clearly.

**Figure 3:**
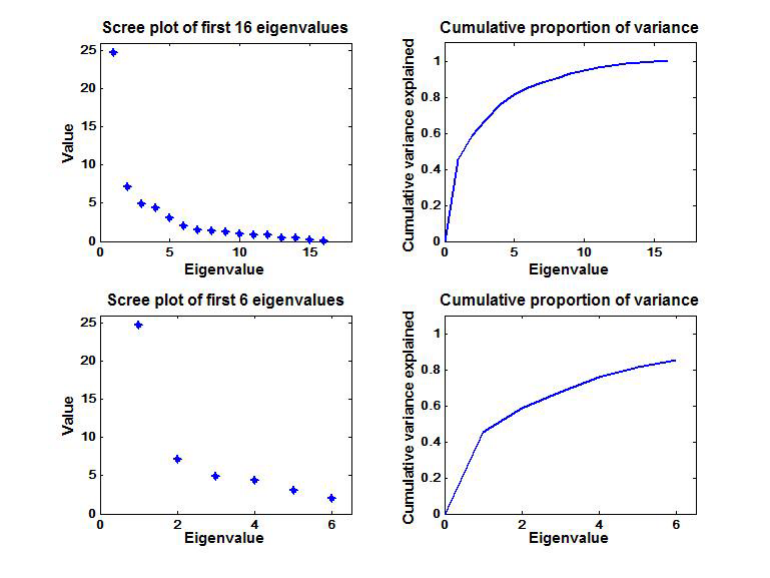
Scree plot of the first 16 and the first 6 principal components.

The scree plot for the first 17 principal components is shown in Figure 3. The first principal component explains 45% of the variance, while the second explains 13% of the variance. Subsequent principal components clearly explain only small proportions of the variance. The first 6 principal components explain 85% of the variability in the standardised data.

**Figure 4:**
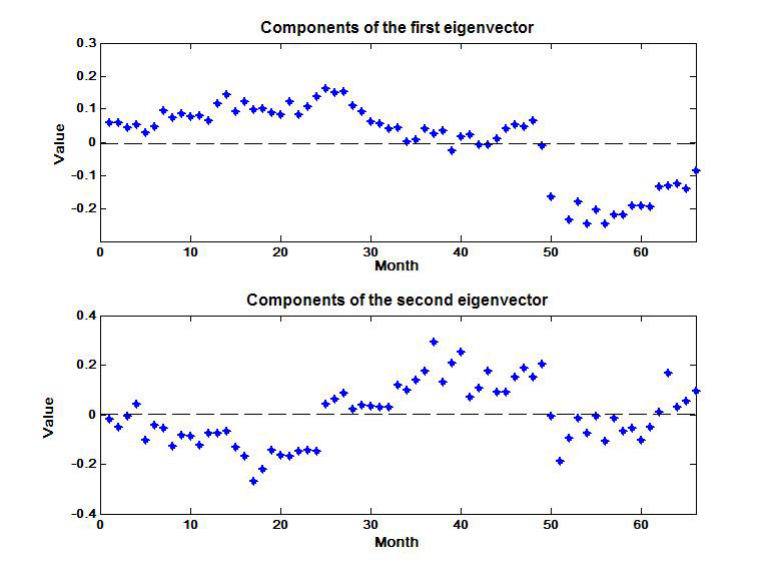
Loadings for the first 2 principal components of the standardised illicit drug market data.

Figure 4 shows the loadings of the 66 months for the first and second principal components.

We assign the loadings into two distinct groups by visual inspection, as shown in Table 2. For principal component 1 there is a run of similar loadings up to month 49, where a jump occurs to a second set of loadings with similar values. This jump represents the point of division of the data into two partitions. Principal component 2 has a run of similar loadings up until month 24, followed by a jump to a new cluster with similar loadings. At month 50 there is another jump, with the loadings after this having similar values to those before month 24. This suggests three partitions, separated from one another by jumps at months 25 and 50.

Depicted graphically (Figure 4), there is clear evidence of clustering in the loadings. Principal component 1 provides a contrast between pre- and post- heroin shortage months, while principal component 2 provides a contrast between those months at the peak of an epidemic cycle (months 25 to 49) and those months at the troughs (the remainder). The structure of these partitions is very clear, with very few observations which cannot be placed immediately. Since principal component 1 explains almost 4 times as much variance as principal component 2, the foremost interpretation of the variation in the data is that obtained from principal component 1. Principal component 2 represents a weaker explanation of the data series' variation. We conclude that in this data set *the contrast between pre- and post-shortage behaviour is of much greater significance than the contrast between peaks and troughs in the data series*. The heroin shortage is clearly shown by PCA to be the single largest structural driver of variability in the data set.

**Figure 5:**
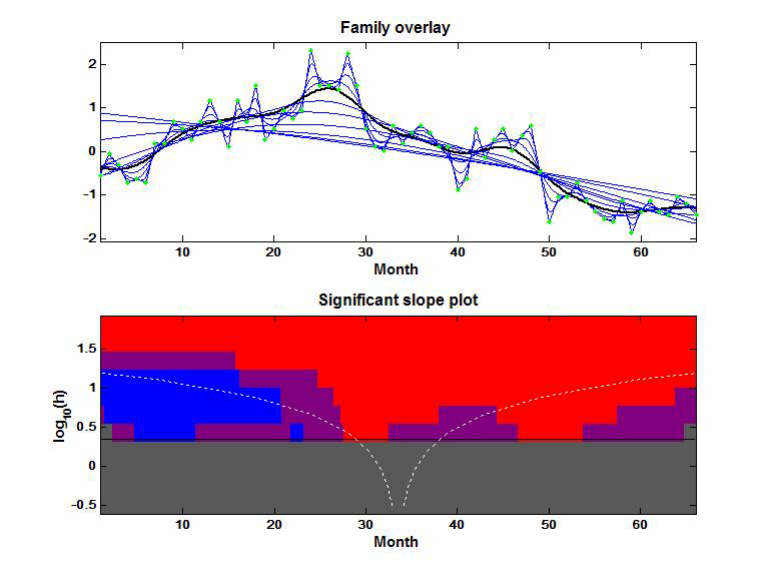
SiZer plots for heroin overdose deaths.

The results of applying SiZer to the heroin overdose death data are shown in Figure 5. The topmost plot gives a 'family overlay' of the smoothed curves for kernel smoothers estimated with a range of different bandwidths, with the thick black curve representing the 'best fit' to these families of smoothers. The lower plot shows regions of statistically significant first derivatives of the smoothers, presented by bandwidth. The 'best fit' smoother is shown as a black line. In this plot regions of statistically significant *increase *in the smoothed curve are shown in blue, areas where the slope is zero are shown in purple, and areas of statistically significant *decrease *are shown in red. The first derivative for the 'best fit' smoother suggests a significant increase in deaths (months 5 – 12), which plateaus (months 12 – 25) and then shows a significant decrease (months 25 – 35). This represents a broad pattern of increase and decrease in a 30 month period between May 1997 and November 1999. There is another significant decrease in deaths between months 48 and 55 (December 2000 and July 2001), corresponding with the point of onset of the heroin shortage. The bandwidth of this best fit is approximately 2 (since log_10_(2) = 0.3), representing a Gaussian kernel calculated in a neighbourhood of approximately 8 months. Larger bandwidths (for example, a bandwidth of 10) show a longer period of increase in the first half of the data series, and a statistically significant decrease for the remainder of the series. The statistically significant decrease between months 48 and 55 is significant at *all bandwidths for which data density is sufficient to estimate significance*. Evidence for the heroin shortage prevails at small bandwidths where the long-term decline in the data series is no longer evident, suggesting it is a separate phenomenon from the 'epidemic' cycle evident at other points of the data series and over wider bandwidths of smoothing.

**Figure 6:**
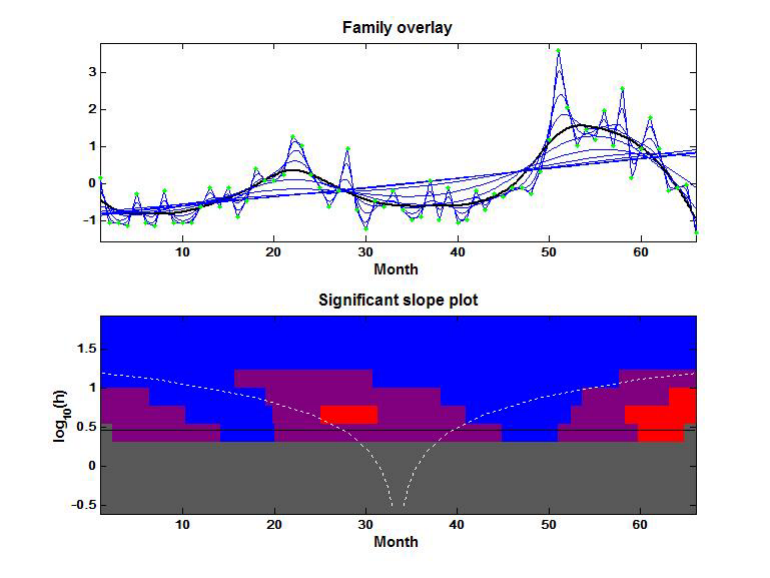
SiZer plot for cocaine possession offences.

Figure 6 shows SiZer plots for the cocaine possession offence data. The 'best fit' smoother suggests the presence of a small increase between months 5 and 12 (May 1997 and December 1997) and a further increase between months 45 and 51 (September 2000 and March 2001), which reverses after month 60. Over larger bandwidths the specific increase between months 45 and 51 disappears, merging into a long period of growth in the data series. Again, the change in the data series at month 49 is evident in as narrow a timeframe as 6–8 months, where it represents one of two expansions in the scale of the market. This expansion reverses within 6–8 months. The smoother suggests two temporary expansions in the market, one between months 5 and 12 which never reverses, and one at the time of the heroin shortage which levels off after 6–8 months and declines 6–8 months later. This pattern of behaviour is more consistent with the observed data than is the observation of a consistent long-term expansion obtained from a view over wider bandwidths.

Both of these SiZer plots provide clear evidence for a sudden change in the period July 2000 – February 2001, observable separately from long-term trends when viewed on a narrow timeframe. The nature of the change and its long term behaviour is consistent with other time series analyses of these data [14] and [5].

## Discussion

Principal component analysis of key indicator data from NSW shows clearly that the first two principal components contain distinct and distinguishable information. The first of these principal components, explaining 45% of the variability in the data, represents a contrast between pre- and post- heroin shortage time points. The second of these, explaining 13% of the variability in the data, represents a contrast between months at the peak of the heroin-market data series (between 1999 and 2001) and months in the 'trough' either side of this peak. The ordering of these principal components, their clear interpretation and the absence of correlation between them is strong evidence for the interpretation of the heroin shortage as an event that occurred separately of long-term cycles in Australian illicit drug markets, and which accounted for a great deal more variability in the data than other variability which happened over a longer term.

It is unlikely that the heroin shortage represented a natural 'correction' to the illicit drug market which occurred after the end of a long period of decline in heroin markets, since the principal component representing the shortage is uncorrelated with the principal component representing the long-term trend in the market, and because the shortage explains four times as much variability in the data as does the phenomenon which it might be supposed to be 'correcting'. The SiZer analysis presents further evidence of the existence and placement of the shortage, and its importance relative to long-term trends in the data. In the two series presented here the period surrounding the heroin shortage is the only period in the data in which a significant change is visible *over all bandwidths for which there is sufficient data to estimate significance*. It is also clearly evident as a separate phenomenon from longer-term trends (such as earlier increases and decreases in the level of the data series) in the 'best fit' smoother obtained from the SiZer.

These analyses, based on the twin methods of extraction of lower-dimensional data on the basis of maximum variability and uncorrelatedness (PCA) and scatterplot smoothing over a wide range of smoothing choice (the SiZer) both support the theory that the heroin shortage was a real, identifiable event in Australian illicit drug markets. The analyses also provide evidence that the heroin shortage which was not directly related to long-term drug market trends and was the single largest phenomenon in the illicit drug market over the 6 year period studied. These findings have several important ramifications for drug market policy. Firstly, to the best of the authors' knowledge, there is no other study which shows that a non-random shock, possibly deliberately induced by drug market factors, can be of considerably greater magnitude than the natural cycles in the market. Secondly, this study confirms mathematically the commonly-held theory that drug markets work in cycles, and gives some evidence of the scale over which this cycle is working in modern Australia (about 6 – 8 years from trough to trough). Although it is likely that more extensive harm reduction policies (such as readily available methadone treatment) and better supply reduction policies (such as high level interdiction activities) may change the long-term patterns in the markets, this finding may help policy-makers to predict future trends in the public health consequences of illicit drug use, and may help inform long-term predictions of numbers of drug users. Incorporation of this scale into historical data on heroin overdose deaths (for example) may enable long-term changes in the scale of the epidemic to be estimated, leading to more accurate future predictions of heroin market movement. Finally, inasmuch as the scale of heroin markets may be related to the numerical behaviour of major indicator data series, drug markets may be subject to non-random influence at their peak as well as when they are heading towards their trough, giving some comfort to those who believe that drug markets are controllable even when they are at their peak

## Conclusion

Existing models of drug markets often suggest that they are beyond government or social control, and that the high rates of death and illness caused by rampant heroin markets at their peak are an inevitable and unavoidable consequence of these markets. The findings of this paper suggest that this is not the case; that even at their peak these markets may be vulnerable to intervention; and that drug market policy settings can be manipulated to prevent unnecessary death and illness in young Australians.

## Competing interests

The author(s) declare that they have no competing interests.

## Authors' contributions

All authors 1) have made substantial contributions to conception and design; 2) have been involved in drafting the manuscript or revising it critically for important intellectual content; and 3) have given final approval of the version to be published.

## Pre-publication history

The pre-publication history for this paper can be accessed here:



## Figures and Tables

**Table 1:** data series used for the study

**Data series**	**Source**
Heroin possession offences	Bureau of Crime Statistics and Research (BOCSAR)
Cocaine possession offences	BOCSAR
Amphetamine possession offences	BOCSAR
Prostitution offences	BOCSAR
Ambulance attendances to heroin overdose	NSW Ambulance Service
Methadone maintenance treatment registrations	Pharmaceutical Services Branch (PSB)
Methadone maintenance treatment new registrations	PSB
Heroin calls of concern	Alcohol and other Drug Information Service (ADIS)
Cocaine calls of concern	ADIS
Amphetamine calls of concern	ADIS
Heroin overdose deaths	Division of Analytic Laboratories
Amphetamine overdose attendances to accident and emergency departments	NSW Health
Robbery without a weapon	BOCSAR
Steal from Motor Vehicle	BOCSAR
Drug psychosis attendances at accident and emergency departments	NSW Health
Robbery with a weapon not a firearm	BOCSAR
Break and enter dwelling	BOCSAR

For more detail on each of these data series see [12]

**Table 2:** months assigned to the partitions for principal components 1 and 2

	**Partition 1**	**Partition 2**	**Partition 3**
**Principal component 1**	All months after month 49	All other months before month 49	-
**Principal component 2**	All months before month 25	All months between 25 and 49	all months between 50 and 61
